# Readmission rates among patients with cirrhosis across different follow-up periods and prevalence of major complications: a systematic review and meta-analysis

**DOI:** 10.1515/med-2026-1488

**Published:** 2026-09-23

**Authors:** Ruiyu Xie, Xiaotong Jing, Minghui Wang

**Affiliations:** Department of Gastroenterology, The Fourth Hospital of Hebei Medical University, Shijiazhuang, China; Department of Hematology, Hebei Key Laboratory of Hematology, The Second Hospital of Hebei Medical University, Shijiazhuang, Hebei, China; Fengtai 12th Retired Cadres Sanatorium Clinic, Beijing Municipal Bureau of Retired Cadre Service, Beijing, China

**Keywords:** cirrhosis, readmission rate, prevalence, systematic review, meta-analysis

## Abstract

**Objectives:**

To estimate pooled readmission rates among adults with cirrhosis at 30 days, 90 days and 1 year after index hospitalization, and to describe the prevalence of major cirrhosis-related complications among 30-day readmitted patients.

**Methods:**

PubMed, Embase, Web of Science and the Cochrane Library were searched from inception to February 7, 2025 without language restrictions. Readmission was defined as rehospitalization after discharge from an index hospitalization, using the definition and ascertainment window reported by each included study. Pooled proportions were estimated using a random-effects model with Freeman-Tukey double-arcsine transformation. Heterogeneity was assessed using the Chi-square test and I^2^ statistic. Sensitivity analyses were conducted, and small-study effects were explored using funnel plots and Egger’s test when sufficient studies were available.

**Results:**

Thirteen cohort studies involving 316,705 patients with cirrhosis were included. The studies were published from 2015 to 2022 and sample sizes ranged from 111 to 134,038 patients. Nine studies reported 30-day readmission rates ranging from 15 % to 46 %, with a pooled estimate of 27 % (95 % CI: 20–35 %; I^2^=100.0 %, heterogeneity p<0.001). Five studies reported 90-day readmission rates ranging from 25 % to 53 %, with a pooled estimate of 34 % (95 % CI: 29–40 %; I^2^=99.6 %, heterogeneity p<0.001). Two studies reported 1-year readmission, with an exploratory pooled estimate of 33 % (95 % CI: 26–39 %; I^2^=97.7 %, heterogeneity p<0.001).

**Conclusions:**

Readmission after hospitalization for cirrhosis is common across short- and longer-term follow-up windows. However, the pooled estimates should be interpreted cautiously because heterogeneity was extreme, definitions varied across studies, and the 1-year estimate was based on only two studies. Ascites was the most frequently reported complication among 30-day readmitted patients, but the available data do not establish that ascites independently increases readmission risk.

## Introduction

Cirrhosis, the terminal stage of chronic liver disease, progresses from a compensated to a decompensated state and is accompanied by major complications, including ascites, hepatic encephalopathy (HE), acute kidney injury-hepatorenal syndrome (AKI-HRS), variceal hemorrhage (VH) and hepatocellular carcinoma (HCC) [1], 2]. Cirrhosis is responsible for substantial mortality worldwide, with a particularly high burden in East Asia and the Pacific region [3]. Alcohol-related liver disease accounts for a large proportion of cirrhosis in many developed countries, whereas viral hepatitis and other etiologies remain important in many developing settings [4]. As cirrhosis prevalence has increased over the past decade [5], its impact on public health and healthcare systems has also grown [6]. The Global Burden of Disease study has shown a major disability-adjusted life-year burden attributable to cirrhosis [7]. In the United States, cirrhosis was associated with approximately 1,000,000 outpatient visits and 325,000 emergency department visits in 2014, with annual healthcare costs estimated at 29.9 billion US dollars [8]. Hospital admissions and readmissions contribute substantially to this economic burden [9], making post-discharge care an important target for quality improvement.

Despite the clinical and economic importance of cirrhosis-related rehospitalization, evidence regarding readmission burden across standardized follow-up windows remains fragmented. Thirty-day readmission is widely used as a short-term quality and transitional-care indicator; 90-day readmission may capture intermediate instability after discharge; and 1-year readmission reflects longer-term disease burden and healthcare utilization. We hypothesized that patients with cirrhosis experience a high burden of readmission across these time windows, but that pooled estimates would vary because of differences in readmission definitions, diagnostic criteria, cirrhosis severity and healthcare systems. Therefore, the research question of this systematic review and meta-analysis was: what are the pooled 30-day, 90-day and 1-year readmission rates among adults with cirrhosis, and what is the prevalence of major cirrhosis-related complications among 30-day readmitted patients?

## Methods

This study was conducted according to the Preferred Reporting Items for Systematic Reviews and Meta-Analyses (PRISMA 2020) statement [10]. The protocol was registered in the International Prospective Register of Systematic Reviews (PROSPERO; registration number: CRD42023397875).

### Search strategy

We searched PubMed, Embase, Web of Science and the Cochrane Library from inception to February 7, 2025 without language restrictions. The Medical Subject Headings (MeSH) terms and keywords were: (“Liver Cirrhosis” OR “Hepatic Cirrhosis” OR “Liver Fibrosis”) AND (“Patient Readmission” OR “Readmission*” OR “Rehospitalization*”). Detailed search strategies are provided in Supplementary Table 1. References of eligible articles and relevant systematic reviews or meta-analyses were also screened.

### Inclusion criteria

Studies were eligible if they met all of the following criteria: (1) observational study design, including cohort, case-control or cross-sectional studies; (2) adult participants (age >18 years) with cirrhosis diagnosed using appropriate clinical criteria or validated International Classification of Diseases (ICD-9 or ICD-10) codes; and (3) reported readmission rates at 30 days, 90 days or 1 year after an index hospitalization, or reported complication data among readmitted patients.

### Exclusion criteria

Studies were excluded if they were conference abstracts, study protocols, case reports, reviews, duplicate publications or reports without relevant readmission outcomes.

### Study selection

Two reviewers (RX and XJ) independently screened titles and abstracts, assessed full texts and selected eligible studies. Disagreements were resolved through discussion with a third reviewer (MW).

### Data extraction

Two reviewers (RX and XJ) independently extracted the following information according to good-practice recommendations for systematic reviews: author, country, publication year, study design, diagnostic criteria for cirrhosis, study period, sample size, sex distribution, liver disease etiology, readmission definition, readmission window, whether planned readmissions were excluded, whether readmissions were liver-related or all-cause, whether death was handled as a competing risk, liver-related complications among readmitted patients and readmission rates [11]. Disagreements were resolved through discussion with a third reviewer (MW).

### Readmission definition and follow-up periods

For this review, readmission was operationally defined as rehospitalization after discharge from an index hospitalization among adults with cirrhosis, using the definition and ascertainment window reported by each included study. We extracted whether readmissions were all-cause or liver-related, planned or unplanned, and whether death was treated as a competing risk. Because these elements were inconsistently reported and could not be harmonized at the aggregate-data level, the pooled estimates should be understood as syntheses of reported readmission rates rather than fully standardized unplanned liver-related readmission rates. The 30-day, 90-day and 1-year windows were selected because they were the most commonly reported time points and have distinct clinical interpretations: early transitional care, intermediate post-discharge instability and longer-term disease burden, respectively.

### Risk of bias assessment

Because all eligible studies were cohort studies, the Newcastle-Ottawa Scale (NOS) was used to assess risk of bias [12]. The NOS evaluates selection, comparability and outcome domains. Studies were classified as low quality (0–3 stars), moderate quality (4–6 stars) or high quality (7–9 stars).

### Statistical analysis

For each follow-up window, pooled readmission rates were estimated using a random-effects model with Freeman-Tukey double-arcsine transformation. Heterogeneity was assessed using Cochran’s Q test and the I^2^ statistic. I^2^ values >50 % or heterogeneity p values <0.10 were considered indicative of substantial heterogeneity. The random-effects model was used to account for between-study variability, but it does not eliminate heterogeneity; therefore, pooled rates were interpreted as approximate summary estimates rather than precise universal rates. Because fewer than 10 studies contributed to each time-window analysis and study-level covariates were incompletely reported, formal meta-regression was not performed. We therefore performed leave-one-out sensitivity analyses where possible and interpreted the pooled estimates with caution. The complication subgroup analysis was defined *a priori* as a descriptive analysis of the prevalence of complications among readmitted patients; it was not designed to compare readmission risk between patients with and without a given complication. Funnel plots and Egger’s regression were used only to explore small-study effects for the 30-day analysis, and no definitive inference about publication bias was drawn under extreme heterogeneity. Analyses were conducted using Stata/MP 14.0, and p<0.05 was considered statistically significant unless otherwise specified.

Ethical approval and informed consent were not required for this systematic review and meta-analysis because all data were extracted from previously published studies and no new individual patient data were collected.

## Results

### Database search and study selection

**Figure 1:**
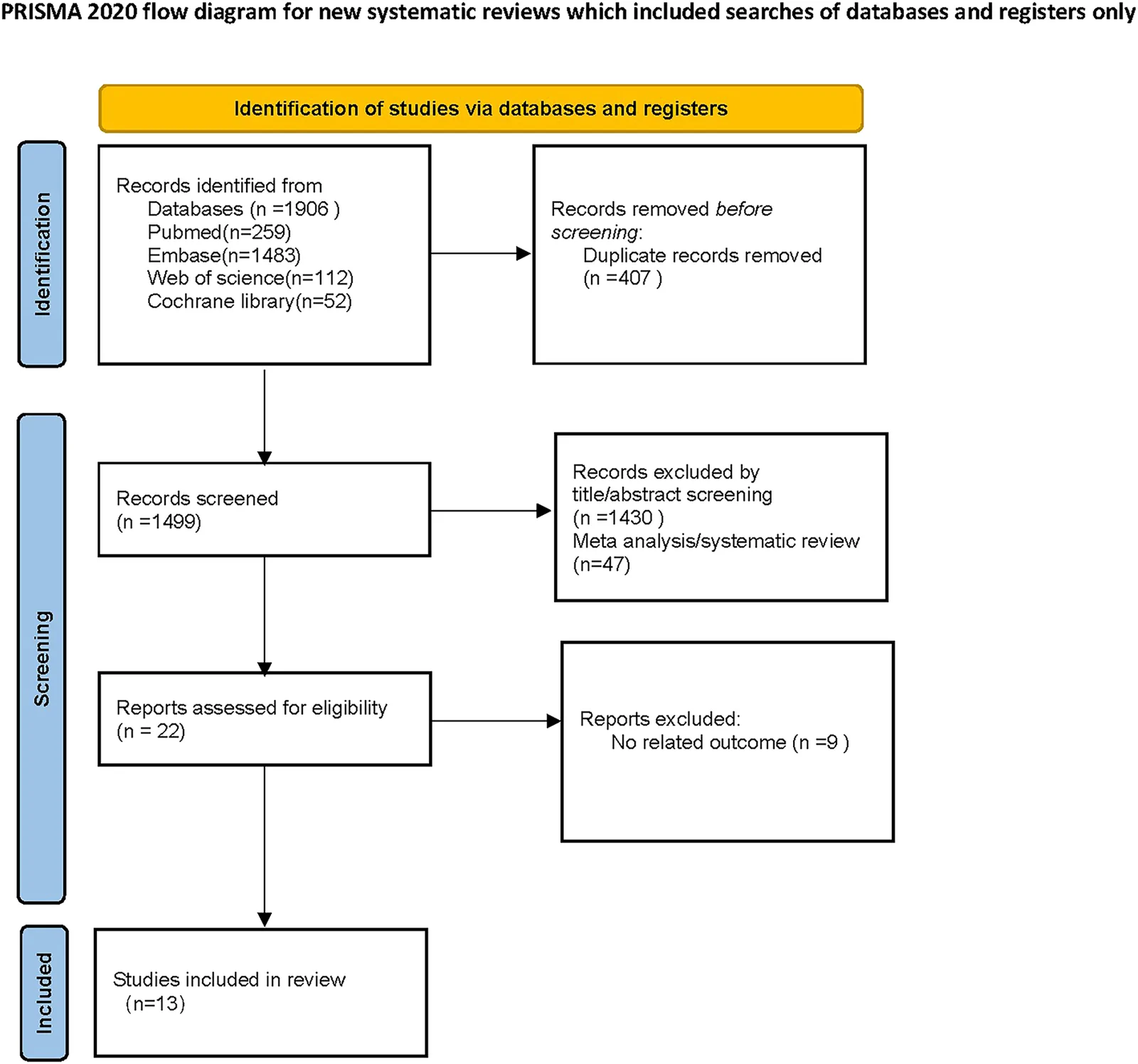
Flow diagram of the study selection process.

A total of 1,906 records were identified through database searching before February 7, 2025. After removal of 407 duplicates, 1,499 records were screened. Of these, 1,477 records were excluded after title and abstract screening, including 47 meta-analyses or systematic reviews. 22 full-text articles were assessed for eligibility, and 13 studies were included in the meta-analysis. The study selection process is shown in Figure 1.

### Study characteristics

Thirteen cohort studies [13], [14], [15], [16], [17], [18], [19], [20], [21], [22], [23], [24], [25] involving 316,705 patients with cirrhosis were included. The studies were published from 2015 to 2022, with sample sizes ranging from 111 to 134,038 patients. Data were collected from six countries: China, the United States, Australia, India, Thailand and Canada. Cirrhosis was diagnosed using validated ICD-9/ICD-10 codes or clinical, imaging, endoscopic or biopsy-based criteria. The included populations varied by geographic region, diagnostic criteria and cirrhosis stage; several studies focused on decompensated cirrhosis, whereas others included broader cirrhosis populations. Because stage-specific data were not consistently available, stratified analyses by cirrhosis stage could not be performed. Baseline characteristics are shown in Table 1.

**Table 1:** Baseline characteristics of included studies.

Reference	Country	Study design	Diagnosis	Study period	Sample size	Age (years)	M/F	Etiology of liver disease	
								Alcohol-related	Non-alcohol-related
Vaz et al. [13]	Australia	Retrospective cohort study	ICD-10 codes	2019.1.1–2019.12.31	179	59 (48–65)	113/66	96 (53 %)	83 (47 %)
Singal et al. [14]	USA	Retrospective cohort study	ICD codes	2016–2017	53,348	(−)	(−)	(−)	(−)
Gajendran et al. [15]	USA	Retrospective cohort study	ICD-9 codes	2014.01–2014.11	57,305	59.93 (11.96)	35,694/21,611	29,553 (51.6 %)	27,752 (48.4 %)
Bajaj et al. [16]	USA	Cohort study	Liver biopsy or clinical/imaging features	2020.03–2020.05	214	(−)	40/81	55 (45.5 %)	66 (54.5 %)
Patel et al. [26]	India	Retrospective cohort study	ICD codes and further clinical and biochemical parameters	2010.01–2017.06	5,138	48.8 ± 14.6	4,085/1,053	2,030 (39.5 %)	3,108 (60.5 %)
Chirapongsathorn et al. [18]	Thailand	Cohort study	ICD-10 codes	2009–2013	134,038	54.20 (13.8)	92,533/41,505	49,594 (37 %)	84,444 (63 %)
Shaheen et al. [19]	Canada	Retrospective cohort study	ICD-9 codes	2014.01.01–2014.09.30	58,954	(−)	36,289/22,665	(−)	(−)
Mukthinuthalapati et al. [20]	USA	Retrospective cohort study	ICD-9 codes	2012.01.01–2013.12.31	733	55 (49–61)	497/236	510 (69 %)	223 (31 %)
Dai et al. [21]	China	Retrospective cohort study	ICD-10 codes	2012.01–2015.12	3,402	(−)	978/2,424	307 (9 %)	3,095 (91 %)
Chirapongsathorn et al. [22]	USA	Retrospective cohort study	ICD-9 codes	2010.1.1–2013.12.31	2,048	60.00	1,200/848	637 (31.1 %)	1,411 (68.9 %)
Seraj et al. [23]	USA	Retrospective cohort study	ICD-9 codes	2011.01.01–2013.12.31	222	56.7 (11.7)	149/73	(−)	(−)
Bajaj et al. [24]	USA	Prospective cohort study	Liver biopsy or clinical/imaging features	2013.12.01–2015.05.01	1,013	(−)	644/369	436 (43 %)	577 (57 %)
Agrawal et al. [25]	USA	Retrospective cohort study	ICD-9 codes	2009.01.01–2013.12.31	111	59 ± 7.8	109/2	81 (72.9 %)	30 (27.1 %)

### Quality assessment

All included studies were assessed using the NOS. The mean NOS score was 8.08, and all studies scored 7 or higher, indicating generally high methodological quality. Detailed scores are shown in Table 2.

**Table 2:** Quality assessment of the cohort studies.

Study	Year	Selection	Comparability	Outcome	Total
Vaz, Tan et al.	2022	****	*	**	7
Singal, A. K., et al.	2022	****	**	***	9
Gajendran, Umapathy et al.	2022	****	**	***	9
Bajaj, J. S., et al.	2021	***	**	**	7
Patel, H., et al.	2020	****	**	**	8
Chirapongsathorn, S., et al.	2020	****	**	***	9
Shaheen, A. A., et al.	2019	****	**	***	9
Mukthinuthalapati, V. V. P. K., et al.	2019	****	**	**	8
Dai, J. Y., et al.	2019	****	**	***	9
Chirapongsathorn, S., et al.	2018	****	**	**	8
Seraj, S. M., et al.	2017	***	**	**	7
Bajaj, J. S., et al.	2016	****	**	**	8
Agrawal, K., et al.	2015	****	**	*	7

### Readmission rate in cirrhosis patients

**Figure 2:**
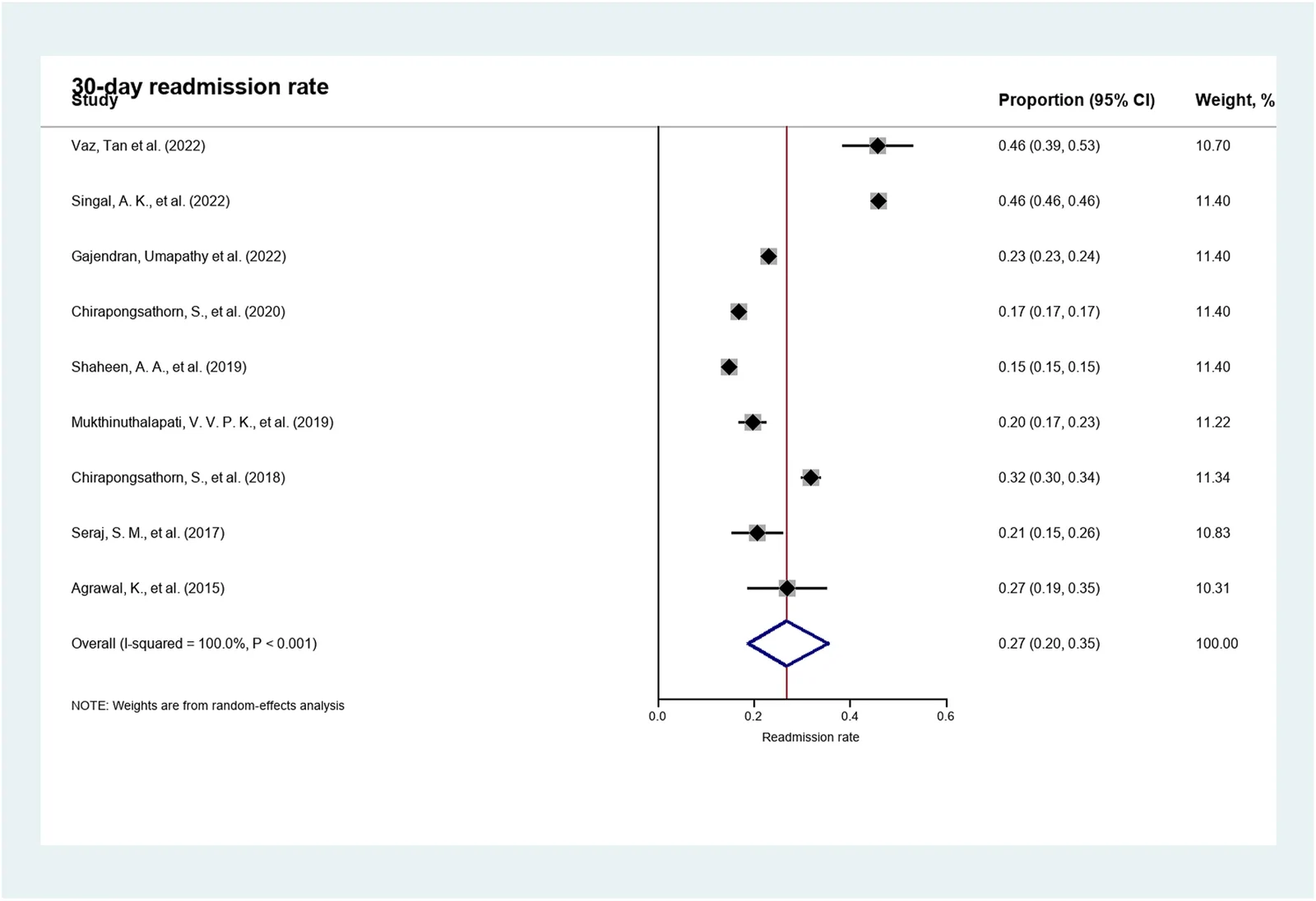
Random-effects meta-analysis of the 30-day readmission rate.

Among the 13 included studies [13], [14], [15], [16], [17], [18], [19], [20], [21], [22], [23], [24], [25], 9 studies [13], [14], [15, [18], [19], [20, 22], 23], 25] reported 30-day readmission rates ranging from 15 % to 46 %. The pooled 30-day readmission rate was 27 % (95 % CI: 20–35 %; I^2^=100.0 %, heterogeneity p<0.001; Figure 2). The wide range and extreme heterogeneity indicate substantial between-study variation rather than statistical stability.

**Figure 3:**
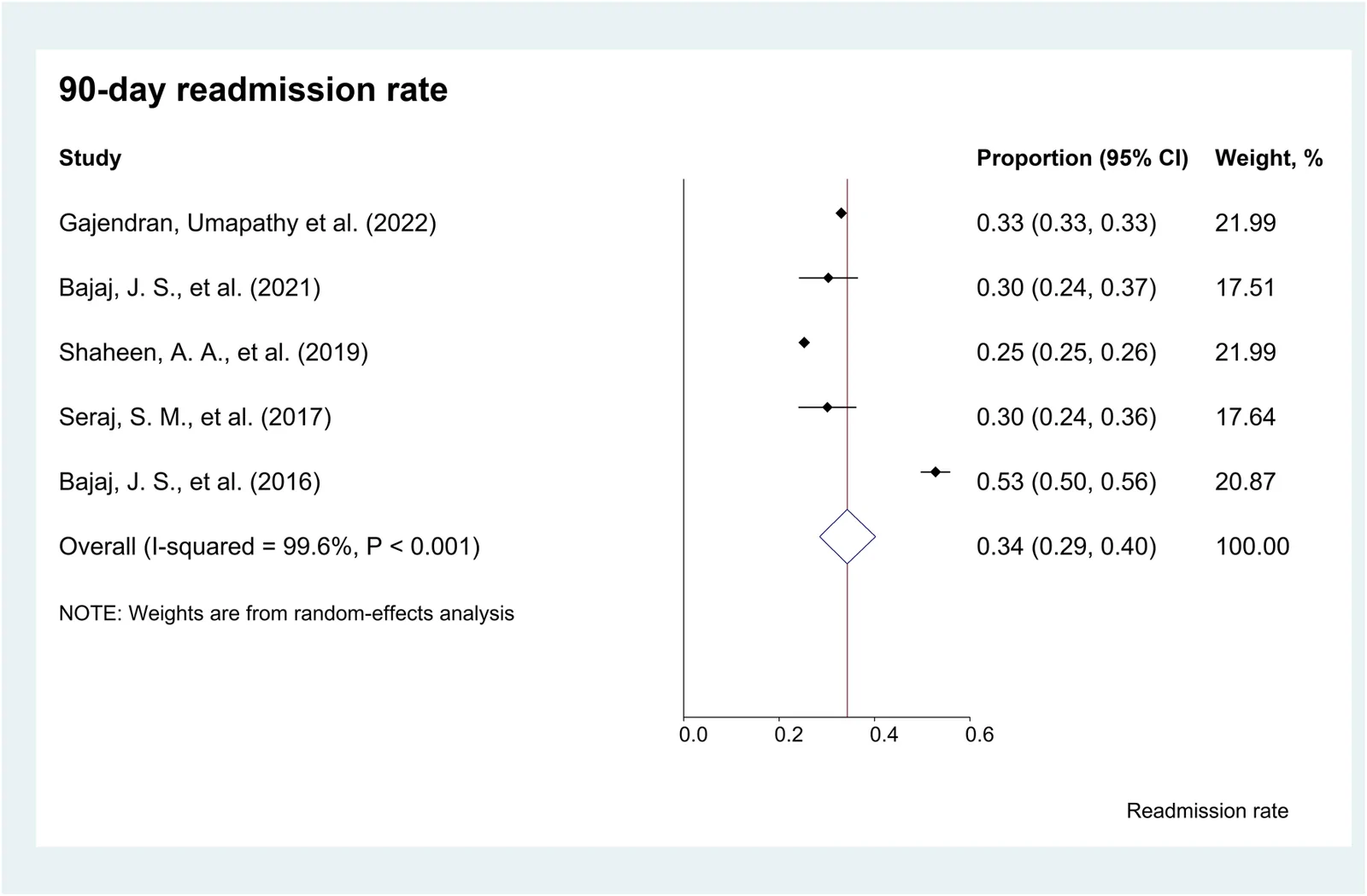
Random-effects meta-analysis of the 90-day readmission rate.

Five studies [15], 16], 19], 23], 24] reported 90-day readmission rates ranging from 25 % to 53 %. The pooled 90-day readmission rate was 34 % (95 % CI: 29–40 %; I^2^=99.6 %, heterogeneity p<0.001; Figure 3). This estimate suggests a persistent intermediate-term rehospitalization burden, but it should be interpreted in light of heterogeneous study populations and definitions.

**Figure 4:**
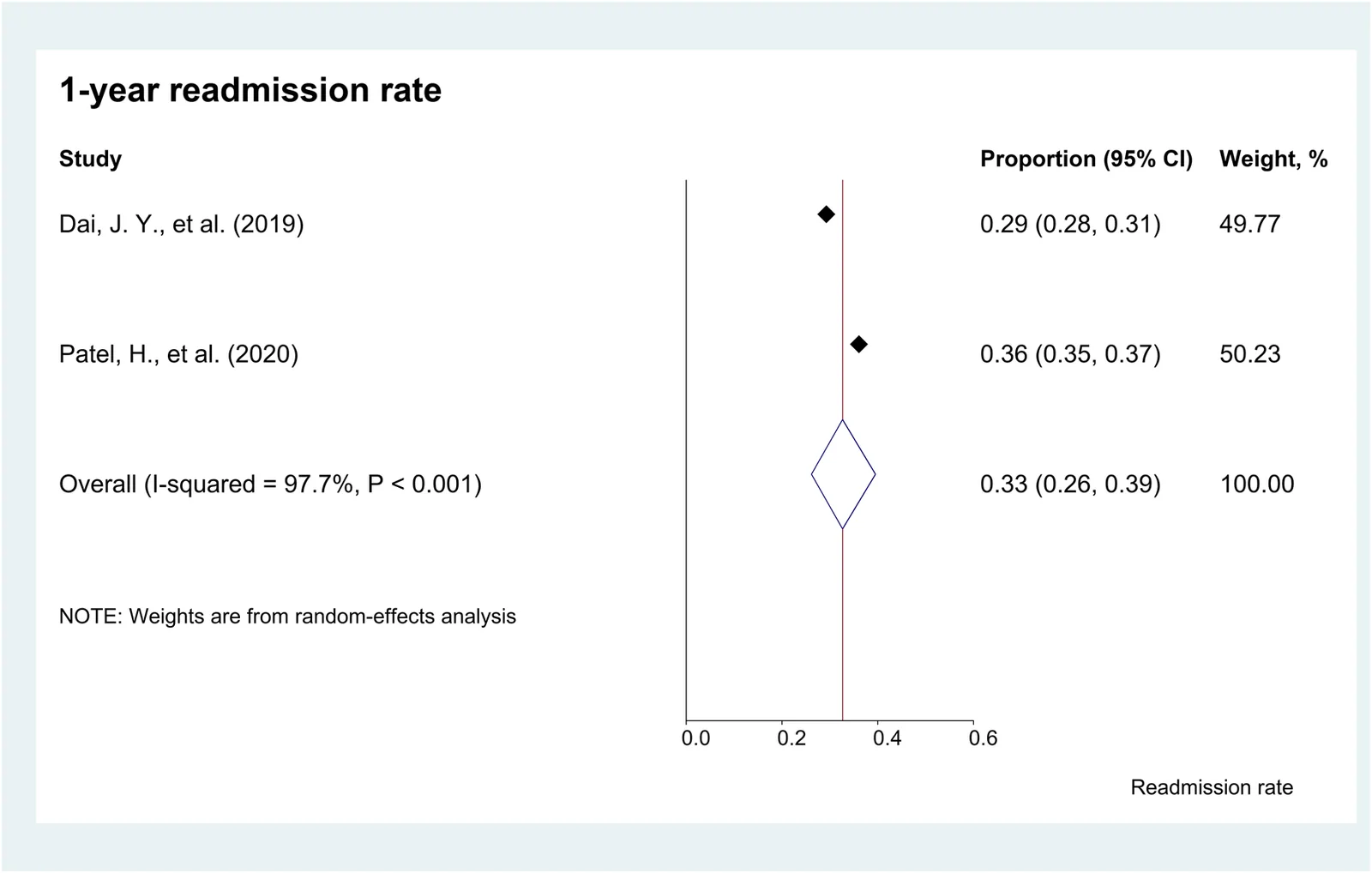
Random-effects meta-analysis of the 1-year readmission rate. The result is exploratory because only two studies were available.

Two studies [17], 21] reported 1-year readmission. The exploratory pooled 1-year readmission rate was 33 % (95 % CI: 26–39 %; I^2^=97.7 %, heterogeneity p<0.001; Figure 4). Because this result was based on only two studies, it was considered statistically underpowered and hypothesis-generating rather than confirmatory.

Leave-one-out sensitivity analyses were performed for the 30-day and 90-day readmission analyses. No single study fully explained the heterogeneity or changed the overall direction of the pooled estimates (Supplementary Figures 1 and 2). Sensitivity analysis was not undertaken for the 1-year estimate because only two studies were available. These findings suggest that the pooled estimates were not driven by one individual study, but they do not eliminate the concern of extreme heterogeneity.

### Subgroup analysis

Descriptive subgroup analyses were conducted for cirrhosis-related complications reported among 30-day readmitted patients, including ascites, HE, AKI-HRS, VH and HCC. Ascites had the highest pooled prevalence among readmitted patients, followed by HE; however, heterogeneity was substantial to extreme across complication estimates (Table 3). These findings describe the complication profile of readmitted patients and should not be interpreted as evidence that patients with ascites have a higher risk of readmission than patients without ascites, because the available aggregate data did not provide the required denominators for such a comparison.

**Table 3:** Descriptive analysis of the prevalence of cirrhosis-related complications among 30-day readmitted patients.

Subgroup	Number of studies	Pooled prevalence among readmitted patients (95 % CI)	I^2^	Interpretation
Ascites	4 [13], 15], 18], 19]	70.0 % (21.5–99.5 %)	100.0 %	Most frequently reported complication; not a risk comparison
HE	4 [13], 15], 18], 19]	44.0 % (11.1–80.3 %)	100.0 %	Common among readmitted patients
AKI-HRS	3 [13], 15], 18]	20.7 % (0.0–65.0 %)	100.0 %	Highly heterogeneous estimate
VH	4 [13], 15], 18], 19]	12.1 % (7.3–17.9 %)	99.6 %	Lower pooled prevalence than ascites/HE
HCC	3 [13], 15], 18]	6.2 % (5.4–7.1 %)	84.0 %	Lowest pooled prevalence

HE, hepatic encephalopathy; AKI-HRS, acute kidney injury-hepatorenal syndrome; VH, variceal hemorrhage; HCC, hepatocellular carcinoma; CI, confidence interval. These estimates describe the prevalence of complications among readmitted patients and do not compare readmission risk between patients with and without each complication.

### Publication bias

**Figure 5:**
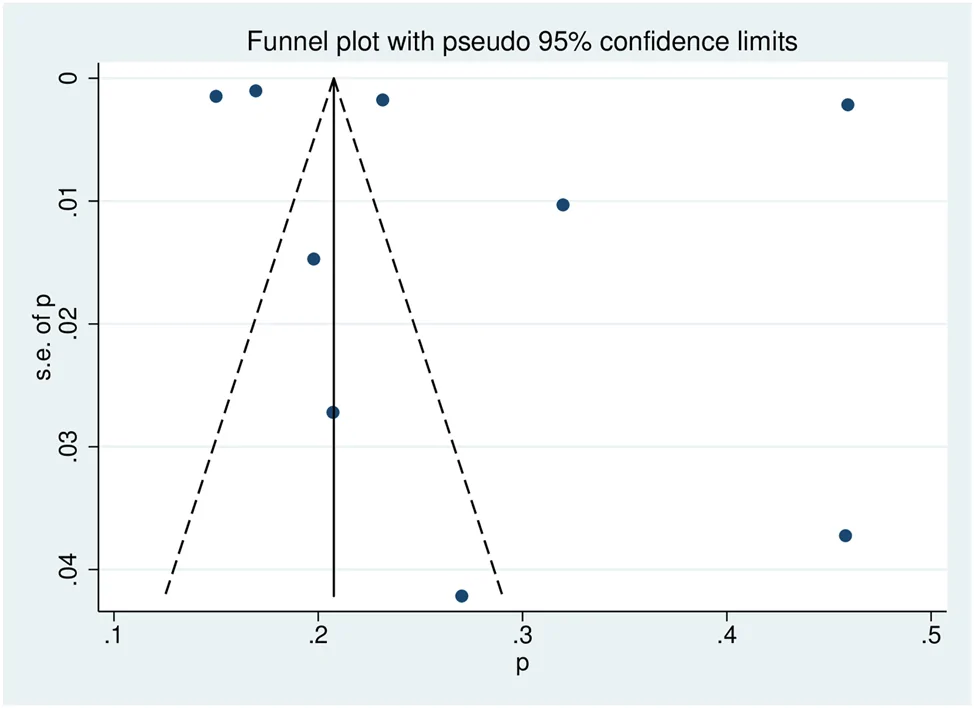
Funnel plot for the 30-day readmission analysis.

The funnel plot for the 30-day readmission analysis appeared asymmetric (Figure 5), whereas Egger’s regression was not statistically significant (p=0.501). Given the extreme heterogeneity and the small number of studies, this assessment should be interpreted cautiously. We therefore do not exclude the possibility of small-study effects or publication bias.

## Discussion

In response to our research question, this systematic review and meta-analysis found that readmission after hospitalization for cirrhosis is common at 30 days and 90 days, while the 1-year estimate remains uncertain because it was based on only two studies. Across 316,705 patients, the pooled readmission estimates were 27 % at 30 days, 34 % at 90 days and 33 % at 1 year. These estimates indicate an important post-discharge burden, but they should be interpreted as approximate summary measures because heterogeneity was extreme for all three time windows.

Our findings are broadly consistent with previous literature. A prior systematic review reported a 30-day readmission rate of approximately 26 % among patients with cirrhosis [27], which is close to the 27 % pooled estimate in the present analysis. A large analysis using the National Readmissions Database reported a 30-day readmission rate of 31.4 % [28]. For the 90-day window, our synthesis supports the concern raised by large North American cohort data that rehospitalization remains frequent beyond the first month after discharge [24]. Evidence for 1-year readmission remains much thinner and appears sensitive to population severity, survival and data-source differences [17], 21]. Compared with studies focused exclusively on 30-day readmission, the present review adds a synthesis of 90-day and 1-year estimates and summarizes the complication profile among 30-day readmitted patients. However, this added scope should be weighed against the limited number of studies for longer follow-up windows and the substantial heterogeneity across data sources.

The high heterogeneity likely reflects differences in healthcare systems, geographic regions, diagnostic criteria, cirrhosis severity, etiology, post-discharge care and readmission definitions. Some studies captured all-cause readmissions, whereas others focused on liver-related readmissions or did not clearly distinguish planned from unplanned readmissions. Death before readmission is a clinically important competing event in cirrhosis and may bias readmission estimates when it is not handled explicitly in the original studies. These issues are particularly relevant when comparing the 90-day and 1-year estimates. The apparently lower 1-year estimate than the 90-day estimate may be explained by the small number of 1-year studies, different baseline severity, survival bias and competing mortality, rather than a true decline in long-term rehospitalization burden.

The descriptive complication analysis showed that ascites was frequently present among 30-day readmitted patients, but this does not establish ascites as an independent predictor of readmission. The high proportion of ascites may reflect its high baseline prevalence in decompensated cirrhosis and its association with complex outpatient management, rather than an independent excess risk of readmission. Such a conclusion would require comparative data on readmission rates among patients with and without ascites, ideally adjusted for cirrhosis severity and competing mortality. Clinically, strategies to reduce readmission should focus on evidence-based management of decompensated cirrhosis, including sodium restriction, appropriate diuretic use, therapeutic paracentesis with albumin when indicated, infection prevention, timely evaluation for transjugular intrahepatic portosystemic shunt or transplantation in selected patients, and structured post-discharge follow-up [29], [30], [31]. Importantly, routine protein restriction should not be recommended, because contemporary nutrition guidance for cirrhosis emphasizes adequate protein intake and prevention of malnutrition, frailty and sarcopenia [32], 33].

## Limitations

This review has several limitations. First, readmission definitions were not standardized across countries, hospitals and administrative data systems; planned admissions, liver-related admission status and competing mortality were incompletely reported. Death before readmission may therefore have biased time-window comparisons when it was not treated as a competing event in the original studies. Second, the included populations spanned compensated and decompensated cirrhosis, different etiologies and different healthcare systems, and stage-specific data were unavailable. Third, heterogeneity was extreme, and the small number of studies precluded robust meta-regression or time-window-specific subgroup analyses, especially for 1-year readmission. Fourth, the complication analyses described proportions among readmitted patients and cannot estimate patient-level risk or causal associations. These limitations mean that the pooled estimates should be used for contextual burden assessment rather than for prediction of individual readmission risk.

## Conclusions

In conclusion, cirrhosis is associated with an important but heterogeneous readmission burden at 30 days and 90 days after hospitalization. The 1-year estimate is based on limited evidence and should be interpreted cautiously. Ascites was common among readmitted patients, but the available data do not prove that ascites independently increases readmission risk. Future studies should use standardized definitions of planned and unplanned readmissions, distinguish liver-related from all-cause readmissions, account for mortality as a competing risk, and report stage-specific data to support more reliable subgroup and meta-regression analyses.

## Supplementary Material

Supplementary Material

Supplementary Material

Supplementary Material
