# Supplementary material for: Readmission rates among patients with cirrhosis across different follow-up periods and prevalence of major complications: a systematic review and meta-analysis
Source: Open Med (Wars). 2026 Sep 23;21(1):20261488. doi: 10.1515/med-2026-1488 (PMC13603070; doi:10.1515/med-2026-1488)
Supplement: Supplementary file 1 [file j_med-2026-1488_suppl_001.docx]

**Supplementary**

**Table 1~3: Details of the Literature Search Strategy**

（1） Pubmed（February 7 2025）

| Search | Query | Results |
| --- | --- | --- |
| 1 | "Liver Cirrhosis"[Mesh] | 100434 |
| 2 | (Hepatic Cirrhosis[Title/Abstract]) OR (Liver Fibrosis[Title/Abstract]) | 24954 |
| 3 | ("Liver Cirrhosis"[Mesh]) OR ((Hepatic Cirrhosis[Title/Abstract]) OR (Liver Fibrosis[Title/Abstract])) | 109559 |
| 4 | "Patient Readmission"[Mesh] | 22284 |
| 5 | (Readmission*[Title/Abstract]) OR (Rehospitalization*[Title/Abstract]) | 48012 |
| 6 | ("Patient Readmission"[Mesh]) OR ((Readmission*[Title/Abstract]) OR (Rehospitalization*[Title/Abstract])) | 53051 |
| 7 | (("Liver Cirrhosis"[Mesh]) OR ((Hepatic Cirrhosis[Title/Abstract]) OR (Liver Fibrosis[Title/Abstract]))) AND (("Patient Readmission"[Mesh]) OR ((Readmission*[Title/Abstract]) OR (Rehospitalization*[Title/Abstract]))) | 259 |

（2） Embase（February 7 2025）

| Search | Query | Results |
| --- | --- | --- |
| 1 | 'liver cirrhosis'/exp | 199949 |
| 2 | 'hepatic cirrhosis':ab,ti OR 'liver fibrosis':ab,ti | 39303 |
| 3 | 'liver cirrhosis'/exp OR ('hepatic cirrhosis':ab,ti OR 'liver fibrosis':ab,ti) | 224745 |
| 4 | 'hospital readmission'/exp | 92136 |
| 5 | readmission*:ab,ti OR rehospitalization*:ab,ti | 87205 |
| 6 | 'hospital readmission'/exp OR (readmission*:ab,ti OR rehospitalization*:ab,ti) | 110583 |
| 7 | ('liver cirrhosis'/exp OR ('hepatic cirrhosis':ab,ti OR 'liver fibrosis':ab,ti)) AND ('hospital readmission'/exp OR (readmission*:ab,ti OR rehospitalization*:ab,ti)) | 1483 |

（3） Cochrane library（February 7 2025）

| Search | Query | Results |
| --- | --- | --- |
| 1 | MeSH descriptor: [Liver Cirrhosis] explode all trees | 3474 |
| 2 | ("Hepatic Cirrhosis"):ti,ab,kw OR ("Liver Fibrosis"):ti,ab,kw (Word variations have been searched) | 1866 |
| 3 | MeSH descriptor: [Patient Readmission] explode all trees | 1370 |
| 4 | (Readmission*):ti,ab,kw OR (Rehospitalization*):ti,ab,kw (Word variations have been searched) | 10241 |
| 5 | #1 OR #2 | 4922 |
| 6 | #3 OR #4 | 10241 |
| 7 | #5 AND #6 | 52 |

（4） Web of Science（February 7 2025）

| Search | Query | Results |
| --- | --- | --- |
| 1 | TS=("Liver Cirrhosis") OR TS=("Hepatic Cirrhosis") OR TS=("Liver Fibrosis") | 62410 |
| 2 | TS=("Patient Readmission") OR TS=(Readmission*) OR TS=(Rehospitalization*) | 51589 |
| 3 | #2 AND #1 | 112 |
